# Factors affecting the quality of life in women with gestational diabetes mellitus: a path analysis model

**DOI:** 10.1186/s12955-020-01293-4

**Published:** 2020-02-18

**Authors:** Soheila Ansarzadeh, Leili Salehi, Zohreh Mahmoodi, Abolfazl Mohammadbeigi

**Affiliations:** 1 Department of Health Education, School of Public Health, Alborz University of Medical Sciences, Karaj, Iran; 2 Department of Health Education and Promotion & Research center for Health, Safety and Environment, Alborz University of Medical Sciences, Karaj, Iran; 3 Department of Health Education & Promotion, School of Public Health, Alborz University of Medical Sciences, P.O. Box 3146-883811, Karaj, Iran; 4 Social Determinants of Health Research Center & School of Nursing and Midwifery, Alborz University of Medical Sciences, Karaj, Iran; 5 Department of Epidemiology & Research Center for Environmental Pollutant, Qom University of Medical Sciences, Qom, Iran

**Keywords:** Gestational diabetes, Quality of life, Path analysis, Iran

## Abstract

**Background:**

Quality of life (QoL) is the most important indicator for assessing the status of health care in chronic diseases. The present study aimed to determine the pathway determinants model of QoL in patients with gestational diabetes mellitus (GDM).

**Methods:**

This cross-sectional study was conducted on 329 women with GM referred to health care centers in Qom, Iran during 2018. Convenience sampling methods was used. Inclusion criteria were: afflicted by GM and received pregnancy care services from health center. Several questionnaires (Knowledge, attitude, self-efficacy (SE), social support (SS), pregnancy distress, self-management(SM) and QoL) were used for data collection. Data were analyzed with SPSS-21 and Lisrel-8.8 software using statistical path analysis.

**Results:**

The mean age of participants was 30.93 ± 5.42 years. The final path model fitted well (CFI =1, RMSEA = 0.0003) and showed that, only age variable from both direct and indirect path had an impact on QoL (B = 0.51). Among variables that directly affected the QoL, SS had the highest effect (B = 1.02) and SE (B = 0.01) had the lowest effect. In the indirect path, only the knowledge variable by affecting the SE had an impact on the QoL (B = 0.0045).

**Conclusion:**

SS had the greatest impact on the QoL. Obviously, providing all the requirements to support patients can help them overcome problems and improve their QoL. Distress negatively affects the QoL through SM and it should be noticed in interventional studies.

## Background

Gestational diabetes (GD) as one of the main metabolic disorders in pregnancy has had an increasing trend in recent years [1]. It refers to glucose intolerance, which is diagnosed for the first time during pregnancy [2]. The disease affects approximately 6% of pregnancies in Iran, with an estimated prevalence of 1. 3% to 18.6% [3]. Other outbreaks have been reported from different countries, varying from 6 to 13% [4].

It is associated with various complications in the mother, fetus and neonatal, among which macrosomia (which is defined as a birth weight over than 4 kg and/or above 90th percentile weight for gestational age or large for gestational age), asphyxia, stillbirth, hypoglycemia, and polycythemia may be present in neonates [5–7]. Preeclampsia, increased incidence of induction and cesarean section (CS), increased chances of developing type 2 diabetes, cardiovascular disease, and increased risk of diabetes in later pregnancies, delayed milk secretion from mammary glands are the common complications in mothers [8–11]. In addition, poor blood glucose control can increase maternal and neonatal mortality rates [12].

Quality of life (QoL) is the most important indicator for assessing the status of health care in chronic diseases [13]. The World Health Organization (WHO) defines QoL as the individuals’ perception of their living conditions in the context of the value system of the surrounding environment [14]. DM affects the QoL in patients [15] and modifies the physical, psychological, and social abilities of patients [16]. QoL in women with gestational diabetes can indicates the different personal response to an appropriate medical treatment.

Identification of factors affecting the QoL in diabetic patients improves the patients’ health and enhances their survival. In this regard, factors, such as diabetic knowledge, attitudes and self-management (SM) are considered as key factors that directly and indirectly affect a QoL in the patients [17]. Factors such as self –efficacy (SE) and social support (SS) are also influenced by SM behaviors, which can affect the QoL of patients [18]. Although, the role of intervening psychosocial factors, such as depression and stress, self-care (SC) behaviors and proper blood glucose control should be considered [19–21].

Few studies have examined the QoL relationships with other variables. For example, the relationship between SM behaviors and QoL [22] SC behaviors and stress [23] Knowledge, attitude and SE [22] SC and knowledge [24] SC behaviors and SS, demographic characteristics and QoL [25] SE, SS and QoL [26]. Therefore, it is necessary to develop a model that can evaluate the direct and indirect effects of these variables on the QoL as well as the relationship between influential variables.

**Fig. 1:**
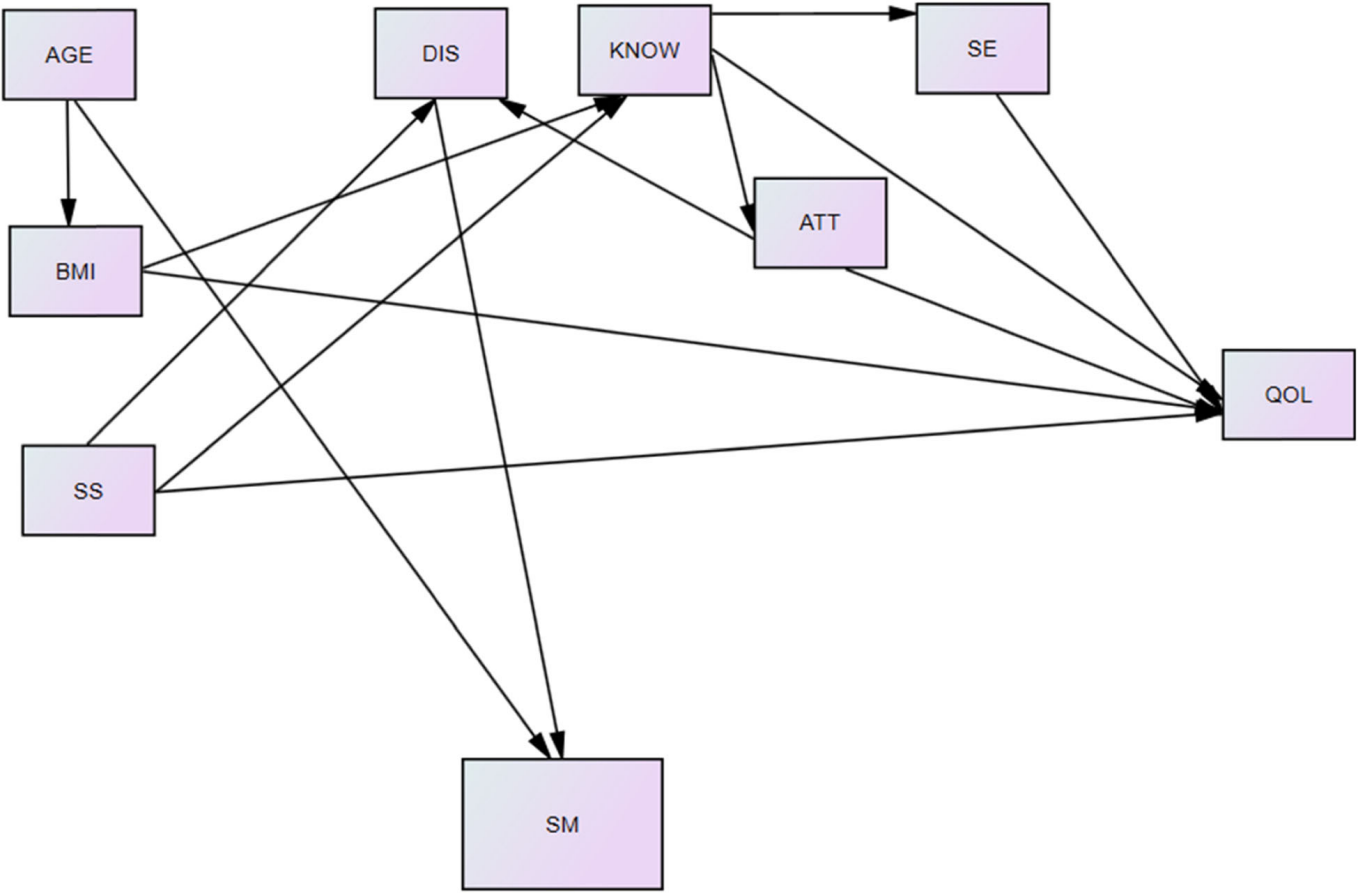
: Proposed path analysis model. Proposal path analysis model of age, BMI, SS (Social support), DIS(distress), KNOW(knowledge), ATT(attitude), SE (Self efficacy), SM (Self-Management) and Qol in patients with gestational diabetes

Therefore, the present study aimed to determine the pathway determinants of QoL in patients with GDM. The proposed model showed association between knowledge, attitude, SE, SS, pregnancy distress, SM and age and body mass index (BMI) with QoL in women with GDM. The proposed model is shown in Fig. 1.

## Methods

### Procedure

This cross-sectional study was conducted on 329 women with GD referred to health care centers in Qom during 2018. First, all health clinics in Qom were identified. Then, we referred to the clinic, and obtained a list of patients, and then the patients with GD were identified. Then, by convenience sampling method, the subjects were selected to complete the sample size. In order to determine the sample size, the following formula was considered with the prevalence of 18% GD based on previous study in Iran [27], 95% confidence interval and precision (d) of 4%.

$$ \mathrm{n}=\frac{{{\mathrm{Z}}^2}_{\left(1-\alpha /2\right)}\mathrm{P}\left(1-\mathrm{P}\right)}{{\mathrm{d}}^2} $$

The incomplete questionnaires were excluded from the study (7% of the questionnaires) and finally 329 questionnaires were analyzed.

The study inclusion criteria were as follows: women who were diagnosed with GD according to the country’s guide, and received pregnancy care services from one of the health care centers in Qom city.

To reach the people, telephone coordination was used and before the research tool was provided, the goals of the study and the willingness of people to participate in the study were evaluated, the written informed consent from the study participants was obtained, and the questionnaire was provided with the necessary explanations. The participants were asked to answer all the questions with accuracy. If participants had any doubts concerning how to fill each part, they were asked to contact the researcher. Some mothers tended to take the questionnaire home and fill it, which allowed them to fill in each questionnaire for about 60 min. The ethics committee of Alborz University of Medical Sciences approved the study (Ethical Code: Abzums.ac.ir.1306.91.)

### Instruments

Several questionnaires were used to collect data.

*1. Demographic characteristics:* include age, marital status, educational level, occupation, ethnicity, pre-pregnancy BMI, midwifery problems, pregnancy history, polycystic ovary syndrome, first-degree relatives, gastrointestinal tract control, and blood glucose control status.

*2. Knowledge:* A questionnaire containing 13 items was used that included 6 items regarding gestational diabetes and its risk factors, 4 items about screening and treatment, and 3 questions about the outcome of the disease in pregnancy that were answered by yes or no. This questionnaire was taken from the Elmurugan & Arounassalame study [28], based on the classification of main designers of the questionnaire; 0–4 scores, indicating low knowledge, 5–8 representing medium, and above 9 representing appropriate knowledge. Validity and internal consistency of this questionnaire was evaluated by content validity and Kuder - Richardson respectively. The Kuder Richardson value was 0.75.

*3. Attitudes:* The attitude questionnaire consisted of 12 items designed according to Anderson et al. [29] questionnaire. The attitude of women with gestational diabetes was assessed about receiving education for diabetes care, seriousness of the disease and glucose control importance. Higher scores represent a more positive attitude. The questionnaire is based on a five- point Likert scale ranging from 1 “completely disagree” to 5 “completely agree”. The content validity was used for the questionnaire validity and Cronbach’s alpha coefficient for internal consistency (it was equal 0.82).

*4. SE:* To assess SE, the Paradly et al. [30] questionnaire was used. According this tool, the participants were asked to list their confidence to achieve certain behaviors related to diabetes control. This questionnaire consisted 35 items was scored based on a five- point likert scale (1. very sure, I cannot do it; 2. Somewhat sure, I cannot do it; 3.not sure, if I can do it; 4. Somewhat sure, I can do it and 5. Very sure, I can do it). In order to calculate the score of SE, the items score was accumulated. Higher scores represent a more SE. The content validity was used for the questionnaire validity and Cronbach’s alpha coefficient for internal consistency (it was equal 0.77).

*5. SS*: The SS questionnaire was used in diabetic individuals for SM. This questionnaire was designed by Naderi Magham et al. [31] and contained 30 questions that were scored based on a five- point likert scale from always (5) to never (1). This instrument includes nutritional subscales (9 questions), physical activity (5 questions), blood glucose monitoring (7 questions), foot care (6 questions) and smoking (3 questions). To calculate the scores at first we scored all items from 1 to 5, second to calculate the row score for each subscale, we added item raw scores and then divide it to number of items in that subscale, third, to transfer row scores to a score ranging from 0 to 100, we used the following formula to calculate the final score: The subscale score = [(subscale row score–1)/4] × 100 [31]. This questionnaire was validated in Iran [31]

*6. Pregnancy Distress:* In this study, pregnancy distress was measured by Tilburg pregnancy distress questionnaire developed by Pop et al. [32] in 2011 and consisted of 16 items and two subscales. The first one is “Negative Affect” and the second is “Social (partner engagement). the first subscale includes 12 items and second subscale includes 4 items. The instrument items were scored based on a 4 -point Likert scale (0. Often, 1: quite often, 2: sometimes, and 3: rarely or never) the scores of 3rd, 5th 6th, 7th, 9th 10th, 11th, 12th, 13th, 14th and 16th items were inversed. The minimum and maximum score is 0 and 48 respectively. The content validity was used for the questionnaire validity and Cranach’s alpha coefficient for internal consistency (it was equal 0.75).

7. SM: SM questionnaire was developed by Schmitt et al. (2013) [33] in 2013. The questionnaire contains 16 questions, which are based on a 4-point Likert scale from 0 (does not apply to me) to 3 (very much apply to me). It includes different areas of SM includes glucose control, physical activity, nutrition, taking the services, and a question that evaluates SM in general. In order to calculate the score of each field, first, its scores were accumulated, then the sum of scores divided by 15 (all of which except the last one), multiplied by 10, thus the score of each field was calculated. This questionnaire was valid based on expert panel views and reliable based on Cronbach’s alpha coefficient. The Cronbach alpha coefficient was 0.83, 0.79, 0.81, and 0.75 for glucose control, physical activity, nutrition and services respectively.

8. *QoL*: The World Health Organization Quality of Life questionnaire (WHOQOL-BREF) [34] was used. The questionnaire contained four subscales (such as physical health, mental health, social relationships, and environmental health) and a general score. This instrument was validated by Nejat et al. in Iran [35]..

For content validity a group of experts (10 specialists) evaluated the questionnaires and for determining the reliability, the Cranach’s alpha coefficient was calculated.

### Data analysis

All data were analyzed by using SPSS software version 21 and LISRELS software version 8. First, the normality of the variables was evaluated using the Kolmogorov–Smirnov test.

The significance correlation between variables was considered as the first hypothesis of path analysis. Eight factors were identified as factors affecting QoL These factors (knowledge, attitude, SE, SS, Pregnancy distress, SM, age and BMI) were considered as independent variables and QoL was considered as a dependent variable.

In order to evaluate the fitness of the model, the fitting index such as × 2/df, RMSEA (Root mean square error of approximation), CFI (Comparative fit index), GFI (Goodness of fit index), NFI (Normal fit index) and IFI (Incremental fit indices) were computed.

## Results

### Characteristics of participants

The mean age of participants was 30.93 ± 5.42 years. The majority of participants (73.0%) had a history of disease in their first degree relatives. Most subjects (68.39%) had wanted pregnancy.

49.24% of subjects were controlled their diabetes by diet, 6.99% by drug and %6.99 by insulin injections. The rest of the subjects used a combination regime (e.g., nutrition and drug, nutrition and insulin, drug and insulin) (Table 1).

**Table 1:** **Demographic Characteristics of Sample study (*n*** **= 329)**

Variable	N (%)
Age	
< 20	6(1.8)
20–25	43(13.1)
26–30	103(31.3)
31–35	110(33.4)
36–40	58(17.6)
> 40	9(2.7)
Marital Status	
Married	322(97.9)
Single	7(2.1)
Occupation	
Governmental	24(7.3)
/non-Governmental	8(2.4)
Housework	297(90.3)
Education	
Illiterate	65(19.75)
High School	207(62.92)
University	57(17.33)
Ethnicity	
Iranian	298(90.58)
Other	31(9.42)
BMI	
> 18.5	6(1.82)
18.24–24.9	86(26.14)
25.9–29	124(37.69)
> 30	113(34.35)
Obstetric Complication	
Yes	111(33.74)
No	218(66.26)
Wanted Pregnancy	
Yes	225(68.39)
No	104(31.61)
PCO	
Yes	29(8.8)
No	300(91.2)
Family History	
Yes	223(67.8)
No	106(32.2)
Glucose Monitoring	
Never	18(5.47)
Sometimes	39(11.85)
Once a week	39(11.85)
Daily	82(24.92)
Physician Recommendation	151(45.90)
Glucose Control	
Nutrition	162(49.24)
Medication	23(6.99)
Insulin	23(6.99)
Mix	121(36.78)

### Relationship between variables

The correlation between variables is shown in Table 2. A significant correlation was found between the variables and QoL varied from 0.14 to 0.79. The strongest and reverse correlation was found between QoL and BMI (Table 2).

**Table 2:** Correlation of study variables (*n* = 329)

	Age	BMI	KNOW	ATT	SE	SS	DISS	SM	QoL
Age	1								
BMI	0.749**	1							
KNOW	−0.169	−0.218*	1						
ATT	−0.128	−0.124	−0.157*	1					
SE	−0.111	−0.184**	0.859**	0.146*	1				
SS	−0.267**	−0.233**	0.096	0.061		1			
DISS	0.135*	0.147**	−0.934**	−0.166*	− 0.853**	−0.101	1		
SM	−0.157*	−0.168*	0.848**	0.133*	0.790**	0.173**	−0.857**	1	
QoL	−0.624**	−0.788**	0.198**	0.086	0.164*	0.203*	−0.159*	0.138*	1

*BMI* Body Mass Index, *KNOW* Knowledge, *ATT* Attitude, *SE* Self Efficacy, *SS* Social Support, *DISS* Distress, *SM* Self-Management, *QoL* Quality of Life

* was significant at level 0.05; ** was significant at level 0,01

### Path analysis model

**Fig. 2:**
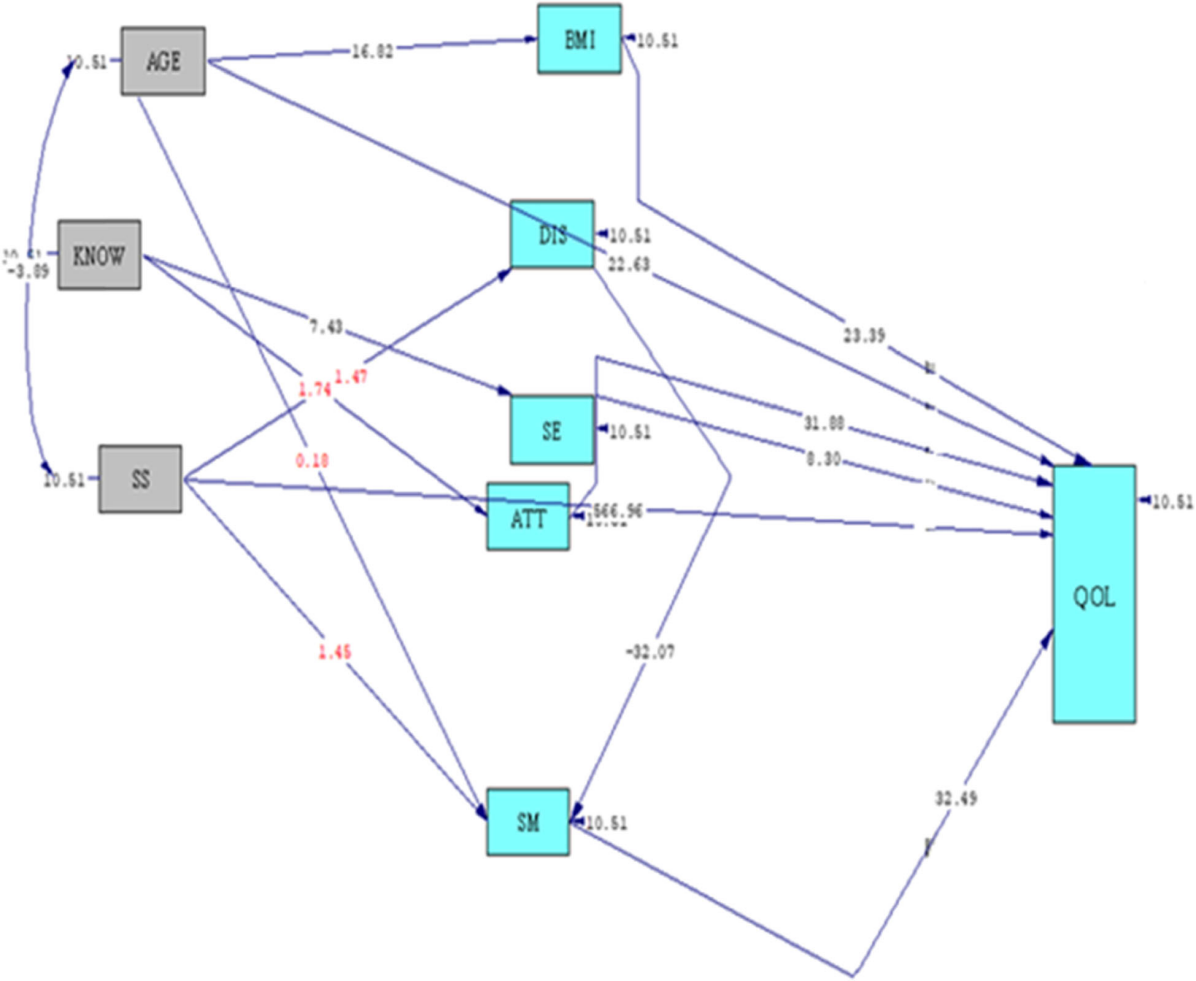
Initial Path Analysis model (based on t-value). Initial path analysis model of age, BMI, knowledge, social support, distress, self efficacy, attitudes, self-management, and QoL in women with gestational diabetes.

The default relationship between the study variables was based on the evidence presented (Fig. 1). Based on existing literature and the correlation between variables, and according to the model indexes, the default model is tested in Fig. 2. Figure 2 shows the significant relationships of the variables based on the results of t value. In pathways that t-value test is less than 1.96 is not significant and is indicated in red on the figure. But in other pathways that the value of t-test is higher than 1.96, the pathway is significant. Accordingly, the pathways analysis, the indirect ways of SS through SM on QoL SS through distress on QoL, and the indirect path of age through SM on QoL were omitted due to insignificance relationships (t-value less 1.96).

**Fig. 3:**
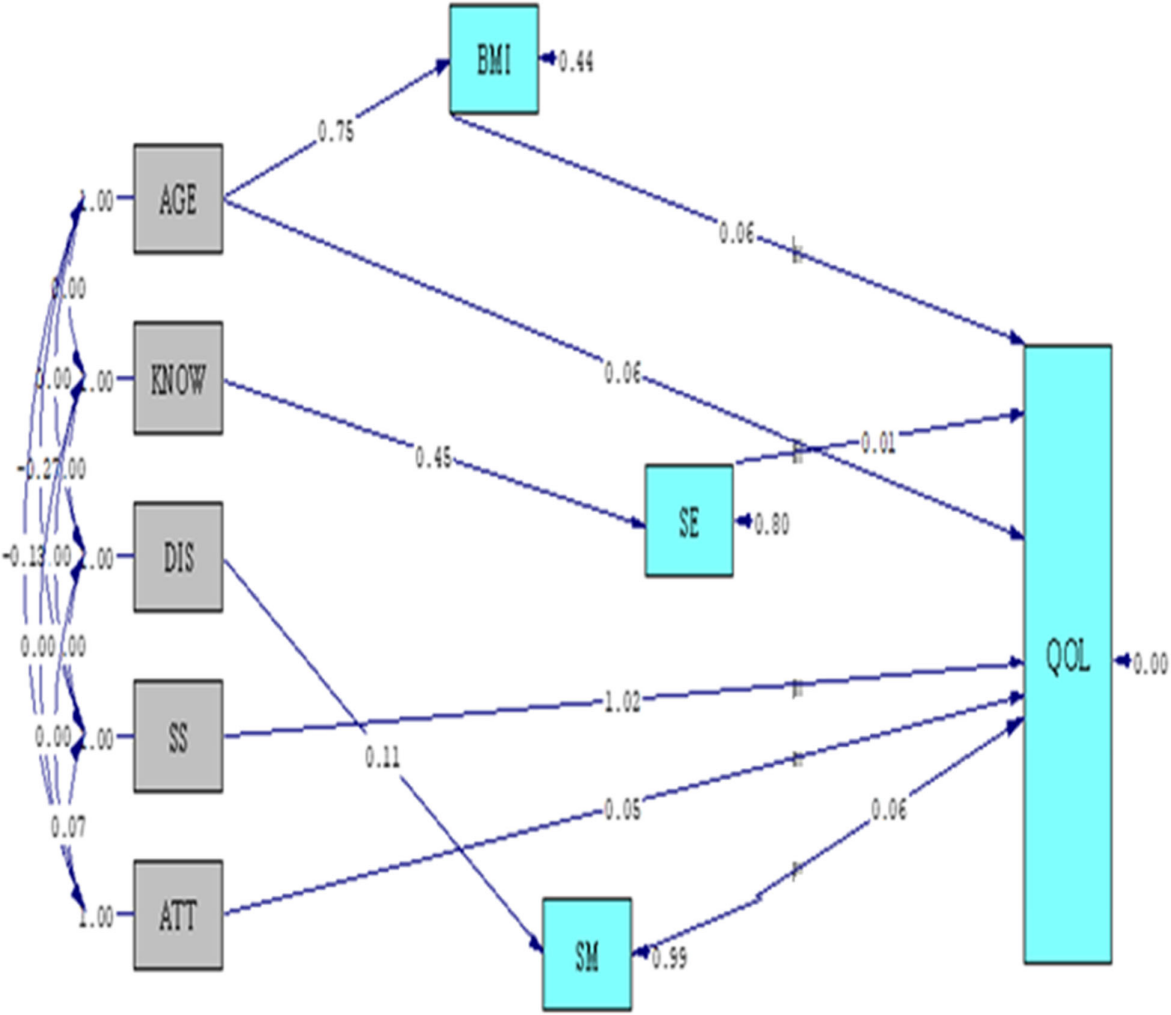
Final Path analysis model. Final path analysis model of age, BMI, knowledge, distress, social support, attitude, self-efficacy, self-management, and QoL in women with gestational diabetes

In Fig. 3, the B standard is specified and non-significant pathways are removed. Based on the final model (Fig. 3), only age variable from both direct and indirect paths through BMI had an impact on Qol (B = 0.51). Among variables that directly affected the QoL, supports had the highest effect (B = 1.02) and SE (B = 0.01) had the lowest effect. In the indirect path, only the knowledge variable by affecting the SE had an impact on the QoL (B = 0.0045) (Table 3).

**Table 3:** Path Coefficients for study Predictors on QoL in patients with gestational diabetes

Predictors	Effects		
	Direct	Indirect	Total
AGE	0.06	0.45	0.51
BMI	0.06		
KNOW		0.0045	0.0045
SS	1.02		1.02
ATT	0.05		0.05
SE	0.01		0.01
SM	0.06		0.06
Diss		0.00066	0.00066

The final path model fitted well (CFI =1, RMSEA = 0.0003, GFI = 0.99), the Goodness of fit Indices for the model indicated in Table 4. The mean and SD of the study variables presented in Table 5. In the present study all variables that entered in the model, were quantitative.

**Table 4:** Goodness of fit Indices for the model

Fitting Index	X^2^	DF	RMSEA	GFI	NFI	CFI	IFI
Model Index	181.14	36	0.0003	0.99	1	1	1
Acceptable Range	X2/df < 5		< 0.05	> 0.9	> 0.9	> 0.9	> 0.9

*IFI* Incremental fit indices, *NFI* Normed-fit index, *GFI* Goodness-of-fit statistic, *RMSEA* Root mean square error of approximation, *× 2* chi-square

**Table 5:** Mean and SD of the study variables

Variables	Mean	SD	Min	Max
Know	7.29	2.014	5	13
Attitude	47	4.27	32	60
SE	106.69	24.29	19.20	158
SS	93.28	22.31	36	149
Distress	21.20	9.34	5	48
SM	14.25	4.06	4	22.01

## Discussion

Based on the final fitted model, age had indirect and direct effects on QoL through BMI in women with GD. The significant negative effects of age on QoL in patients with diabetes are also shown in other studies [36–38]. Evidence shows an increase in undesirable side effects and improper pregnancy outcomes with increasing age which can affect QoL [39–41].

Obviously, the existence of diabetes along with pregnancy increase adverse outcomes of aging in this period, leading to further loss of QoL.

In this study, age also indirectly influences the quality of life through impact on BMI. Higher BMI in pre-pregnancy period is associated with a higher rate of abnormal glucose tolerance [42] which can affect the QoL. Salehi et al. study also showed a significant correlation between BMI and QoL [43].

Based on the results of this study, among variables that directly influence on QoL, SS had the greatest impact on the QoL in women with GD. SS during pregnancy are a protective factor in mothers and help them cope with stressful events in life. This factor during pregnancy not only affects the health of mothers but also pregnancy outcomes. The management of diabetes during pregnancy is identified as a stressful event [44].

Due to the results of this study, pregnancy distress also influenced the QoL through SM. Based on the results of the current study, to improve the QoL in women with GD; patients should overcome the pregnancy distress and accomplish SM behaviors. The study of Razee et al. [45] showed that the ability of women with GD to follow a healthy lifestyle depends on their mental health, social and cultural support.

The results of the current study indicated that knowledge had indirect effects on QoL through SE in women with GD. Other studies have also shown that, in diabetes patients, knowledge alone does not initiate health promotion behaviors [46, 47]. Regarding diabetes SM, there was a consensus that patients should be trained to take care of themselves, and not only knowledge but the ability to manage the disease is necessary to control the disease [48].

SE was another variable that directly affected the QoL. SE plays a key role in the ability of people. Bandura defines SE as a person’s belief in his ability to perform specific behavior. Yale (2015) stated that SE in diabetic patients is one of the predictors of the SC behaviors in them [49]. Therefore, we should pay attention to improve SE in health promotion interventions. We can use appropriate role modeling, verbal persuasion, and dividing tasks into smaller steps. In line with the results of the current study. Moheby et al. [50] demonstrated that the SE stimulates motivation in diabetic patients and has a direct impact on health promoting behaviors. Mrovati et al. also indicated that SE alone explains 38% of the variance in SC behaviors [51].

It is clear that SC behaviors are associated with a higher QoL in patients. Based on the results of this study, SC behaviors had a significant effect on QoL. Similar to many chronic diseases, diabetic patients require both continuous management of their disease and the proper SC behaviors. SC behaviors have significant impact on QoL in diabetic patients [52]. Babazadeh et al. (2017) showed that SC behaviors are the essential component of controlling the disease and improving the QoL in patients with diabetes [21].

Knowledge was another variable which indirectly affected on QoL through SE [53]. Knowledge is considering as one of an important resource for avoiding complications and improving QoL in diabetic patients [23]. Due to Bohanny et al. study diabetic knowledge, obtaining diabetes education and employment status explained 11.8% of the variance in SE [54]. In the current study, 90.3% of the subjects were house worker.

Contrary to Kueh et al. results [17], there was no statistically significant relationship between knowledge and attitude and they were eliminated from the final fitted model. However, attitude directly influenced QoL, and knowledge had an impact on the QoL through SE. Ardena et al. (2010) showed that Knowledge alone is not enough to change lifestyle and improve QoL in diabetic patients, but requires psychosocial factors, such as attitude and SE [55]. There is a significant and positive relationship between attitude and QoL in diabetic patients.

This study had some strengths and limitations; one of the major strengths was the fact that to the best of our knowledge, this is the first study that investigates the direct and indirect effects of the variables on the QoL among pregnant woman with gestational diabetes. Convenience sampling method is one of the limitations of this study. It may lead to bias in the conclusion and generalization of the results of this study. Given that this study carried out among Iranian patients, the results study might not be generalized to all pregnant women with gestational diabetes.

## Conclusion

SS had the greatest impact on the QoL in women with GD. Obviously, providing all the requirements to support patients with GD can help them overcome problems and improve their QoL. Also, distress is one of the factors that negatively affect the QoL through SM behaviors. A variety of therapeutic and supportive methods to reduce distress in patients can be used to enhance SM behaviors and improve the QoL in them.

## Data Availability

The current study datasets and analysis sheets are available and will be provided due to reasonable request.

## Abbreviations

ATT: Attitude
BMI: Body mass index
CFI: Comparative fit index
DISS: Distress
GDM: Gestational diabetes mellitus
GFI: Goodness of fit index
IFI: Incremental fit indices
KNOW: Knowledge
NFI: Normal fit index
QoL: Quality of life
RMSEA: Root mean square error of approximation
SC: Self-care
SE: Self efficacy
SM: Self-management
SS: Social support
